# Fifty-four novel mutations in the *NF1* gene and integrated analyses of the mutations that modulate splicing

**DOI:** 10.3892/ijmm.2014.1756

**Published:** 2014-04-24

**Authors:** WEIHONG XU, XIAO YANG, XIAOXIA HU, SHIBO LI

**Affiliations:** Genetics Laboratory, Department of Pediatrics, University of Oklahoma Health Sciences Center, Oklahoma City, OK 73117, USA

**Keywords:** neurofibromatosis 1 gene, mutation, splicing error

## Abstract

Neurofibromatosis type 1 (NF1) is a common autosomal dominant genetic disorder caused by mutations in the *NF1* gene. One of the hallmarks of NF1 is the high mutation rate in this gene. In this study, we present 127 different *NF1* mutations and 54 novel mutations detected at both the genomic DNA and mRNA level using a retrospective case series review. We found that 25.2% of these different mutations induced aberrant splicing. Of note, 40.6% of these splicing errors were caused by exonic variants. In addition, one mutation produced mosaicism in the post-transcriptional profile. However, studies investigating these splicing aberrations are limited. In order to better understand the pathogenicity of NF1 and to provide a more accurate interpretation in molecular diagnostic testing, combined computational analyses were employed to elucidate the underlying mechanisms of the variants modulating *NF1* gene splicing.

## Introduction

Neurofibromatosis type 1 (NF1) (OMIM 162200) is a progressive autosomal dominant inherited disease and is one of the most widespread genetic disorders worldwide with a prevalence of 1 in 2500- to -3000 live births (1). The clinical characteristics in the NF1 diagnostic criteria include café-au-lait spots, neurofibromas, Lisch nodules, intertriginous freckling, typical osseous lesions and optic pathway gliomas (2). At least 78% of patients who fulfill the NIH diagnostic criteria for NF1 have *NF1* gene mutations (3). Moreover, 5–10% of the cases are caused by a deletion in the *NF1* gene (4). However, the positive rates of the *NF1* mutation findings in clinical diagnostic laboratories vary considerably according to the proportions of the samples from clinically definite or suspected patients. *NF1* is located on 17q11.2 and spans 28,2751 bp in length. This gene contains 60 exons and encodes neurofibromin, a key component in the RAS-MAPK signaling pathway. The RAS-MAPK pathway regulates the proliferation and differentiation of neuronal cells and myocytes (5). Neurofibromin functions as an inhibitor of RAS activation and as a tumor suppressor with a central region that is homologous to RAS-GTPase activation proteins (GAPs) (6). Mutations in the *NF1* gene cause a loss in neurofibromin function, resulting in downstream cell growth activation (7–9). Previous studies have reported over 1,400 different mutations due to the high mutation rate of the *NF1* gene. A high number of these mutations arise as novel mutations; however, there is no hot spot for the pathogenic variations of NF1 (3,10,11).

In this study, we performed a retrospective review of 378 cases and compiled the mutations identified at both the genomic and mRNA level. We present 127 different mutations of the *NF1* gene; 54 of which are novel mutations. In addition, the deletion of the *NF1* gene was detected in 5 cases using fluorescence *in situ* hybridization (FISH) or the comparative genomic hybridization (CGH) array method. With the advent of the genomic DNA and cDNA sequencing approach, splicing abnormalities caused by exonic variants were captured and presented in our data. In addition, 7 of these 13 exonic mutations were novel. Of note, one of these mutations, c.3362A>G, produced mosaicism of a point mutation and mutant exon skipping at the mRNA level.

Accurate splicing of pre-mRNA is not only controlled by the 5′/3′ splice sites (ss), but also by other cis-acting elements, as well as trans-acting factors, i.e., SR proteins and heterogeneous nuclear ribonucleoproteins (hnRNPs). These cis-acting elements generally include the splicing enhancers related to exon-inclusion enhancement, splicing silencers related to exon-inclusion inhibition, the intronic branch point and the polypyrimidine tract (12,13). Although the *NF1* mutation spectrum continues to expand, studies investigating these splicing aberrations are limited (14–18). Thus, integrated analyses using the bioinformatics tools were further applied to provide insight into the mechanisms of these splicing defects caused by exonic variants, as well as other intronic variants at non-consensus splice sites.

## Patients and methods

### Patients

A total of 378 cases were referred for *NF1* gene testing in our laboratory from January, 2006 to May, 2013 and were recruited in this study. The subjects consisted of 338 unrelated probands with clinically definite or suspected NF1 diagnosis and 40 family members. Consent forms were signed by the patients or authorized representatives. All cases underwent a *NF1* gene-sequencing test developed in our laboratory, which was approved by the Ethics Committees at the University of Oklahoma Health Sciences Center, Oklahoma City, OK, USA.

### Mutation screening by Sanger sequencing

Genomic DNA was isolated from peripheral blood samples of the patients using the QIAamp DNA Mini kit (Qiagen, Valencia, CA, USA). mRNA was isolated from the peripheral blood samples using the QIAamp RNA Blood Mini kit (Qiagen). First-strand cDNA was reverse-transcribed using the SuperScript III Reverse Transcriptase kit and random primers (both from Invitrogen, Carlsbad, CA, USA). PCR was performed using specific primers targeting the mRNA coding region of the *NF1* gene. For confirmation, exon-specific genomic DNA sequencing was also performed using specific primers. Primer information will be provided upon request. Sanger sequencing was performed using the BigDye Terminator v3.1 Cycle Sequencing kit (Life Technologies, Foster City, CA, USA) and an ABI 3130xl genetic analyzer (Life Technologies). Sequences were analyzed using Mutation Surveyor software (SoftGenetics, State College, PA, USA).

### In silico analysis

Splice Site Prediction by Neural Network (SSPNN; www.fruitfly.org/seq_tools/splice.html) and the Human Splicing Finder (HSF; www.umd.be/HSF/) were used to investigate the mechanisms of the splicing abnormalities caused by the mutations at the non-consensus splice sites. HSF contains its own programs and other prediction platforms, including the exonic splicing enhancer (ESE) finder (http://rulai.cshl.edu/cgi-bin/tools/ESE3/esefinder.cgi?process=home), RESCUE-ESE (http://genes.mit.edu/burgelab/rescue-ese), FAS-ESS (http://genes.mit.edu/fas-ess), Putative Exonic Splicing Enhancers/Silencers (PESX) designed by Zhang and Chasin (30) and splicing silencer motifs designed in the study by Sironi *et al* (19). This assessment system contains 2 sets (HSF and SSPNN) to examine the potential splice sites, where 1 set (HSF) was used for the potential branch points, 4 sets (PESx, RESCUE-ESE, ESE finder and HSF) for the ESE and 3 sets (PESx, FAS-ESS and splicing silencer motifs) for the exonic splicing silencer (ESS). The query sequences were obtained from the normal and mutated sequences. The SSPNN, HSF and ESE finder provided the scores to value the strength of the splicing-relative sequence motifs using corresponding weight matrices. PolyPhen2 (http://genetics.bwh.harvard.edu/pph2) and SIFT (http://sift.bii.a-star.edu.sg) were applied to predict the potential effect of an amino acid substitution on the structure and function of the NF1 protein. BLASTP was used to align the NF1 protein sequences from the multiple species, including human, chimpanzee, gorilla, cat, dog, mouse, rat, cattle, chicken and zebrafish.

### Real-time PCR

The alternative post-transcriptional profiles in one specific case were produced using the SYBR Master Mix (Life Technologies) and ABI PRISM 7000 Sequencing Detection System (Life Technologies). The *ACTB* gene encoding β-actin was used for normalization. For *ACTB*, the forward primer was 5′-AGCTCCTCCCTGGAGAAGAG-3′ and the reverse primer was 5′-AGCACTGTGTTGGCGTACA-3′. For *NF1*, the forward primer was 5′-GATGTAAAATGTCTTACAAG-3′ and the reverse primer was 5′-CTGCCACCTGTTTGCGCACT-3′. Amplicons targeting on the *NF1* and *ACTB* genes were confirmed using Sanger sequencing. Real-time PCR was performed in triplicate using 5, 2.5, 1.25 and 0.625 ng cDNA.

## Results

### Mutation spectrum

Mutational screening of the *NF1* gene was performed on samples obtained from 378 clinically diagnosed or individuals suspected of having NF1. The mutation nomenclature was based on the NCBI reference NM_000267.3. The exon number was given according to the conventional rule used in the NF1 testing community and previous literatures (10,11,17,18). The mutations were confirmed using the Biobase (HGMD professional version database) and Leiden Open Variation Database (LOVD) to determine the recurrence. In addition, the missense mutations were searched in 1000 Genomes project, dbSNP and Exome Variant Server to rule out normal variants. *NF1* mutations were identified in 169 out of 378 cases; 127 different mutations were observed (Tables I and II). Of these mutations, 54 mutations were novel, of which, 23 were frameshift mutations, 10 were splicing defects, 7 were nonsense mutations and 14 were missense mutations (Table III). The mutations affected almost all exons apart from exon 4c, 14, 23.1, 35, 38 and 49 in the mutation spectrum of the patients, which was consistent with the finding of no hot spot mutations in previous studies (3,10,11). The nonsense mutations were the most common molecular defects found in this study (33/127), followed by the splice-site mutations (32/127) and missense mutations (27/127). In the group of frameshift mutations, deletion (n=23) was prone to occurring compared to insertion/duplication (n=8) and indels (n=4), but most of the insertion/duplication and all of the indels were novel mutations.

Only 50% of the splicing defects disrupted the conserved GT/AG or AT/AC dinucleotides of the splice sites in this study (Table II). By contrast, 3 intronic mutations at non-consensus splice sites (Table IV, subgroup I) generated cryptic 5′ss or 3′ss, resulting in the insertion into the mRNA. Thirteen exonic variants, that were 40.6% of the splicing defects, induced exon skipping or aberrant exons instead of a point mutation based on the genomic DNA and cDNA sequencing methods. Among these exonic mutations, c.1466A>G and c.1885G>A (Table IV, subgroup II) induced aberrant exons with a deletion by generating cryptic 5′ss or 3′ss, respectively. In particular, the 1185G>A mutation was a silent mutation at the genomic DNA level. The other 4 exonic sequence alterations, which resulted in exon skipping, were identified as substitutions at the last nucleotide position of exon 8, 20 and 29, and the last second nucleotide of exon 11 (Table IV, subgroup III). The remaining 7 exonic mutations, which caused exon skipping, did not perturb the natural 3′/5′ss or create cryptic splice sites (Table IV, subgroup IV).

**Figure 1:**
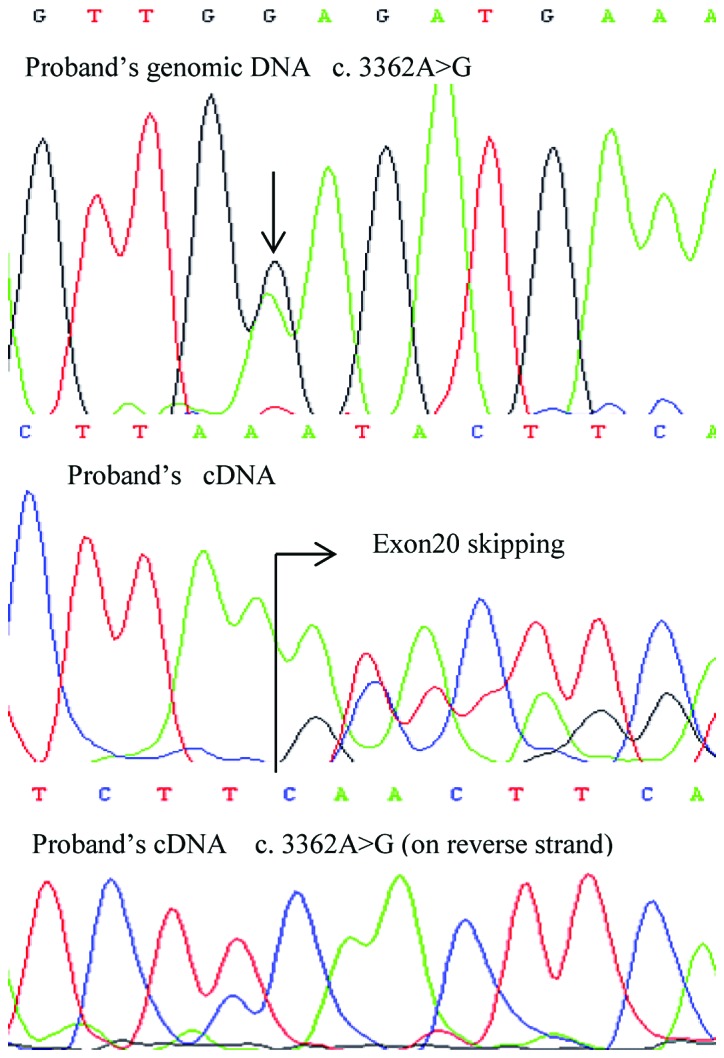
The Sanger sequencing data showed a germline mutation (c.3362A>G) resulting in the missense mutation, E1121G, and exon 20 skipping coexisting at the mRNA level.

**Figure 2:**
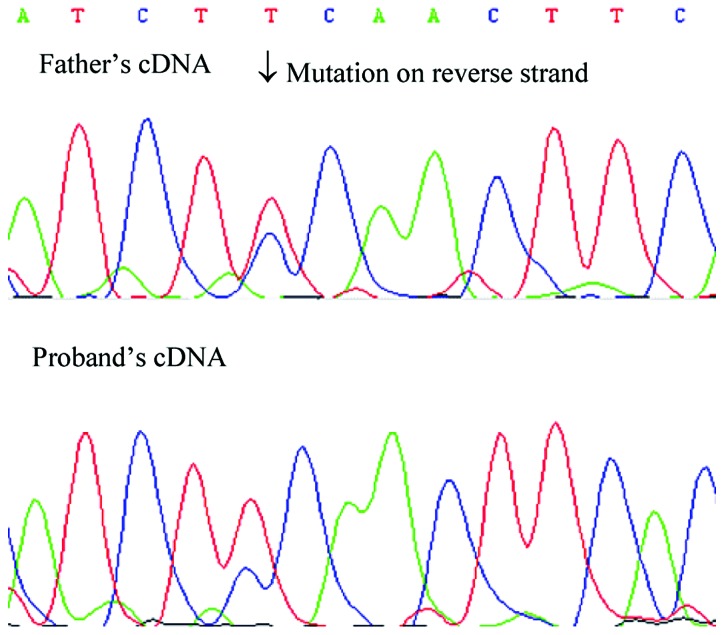
Comparison of the signal strength of the missense mutation in the mosaic cases.

**Figure 3:**
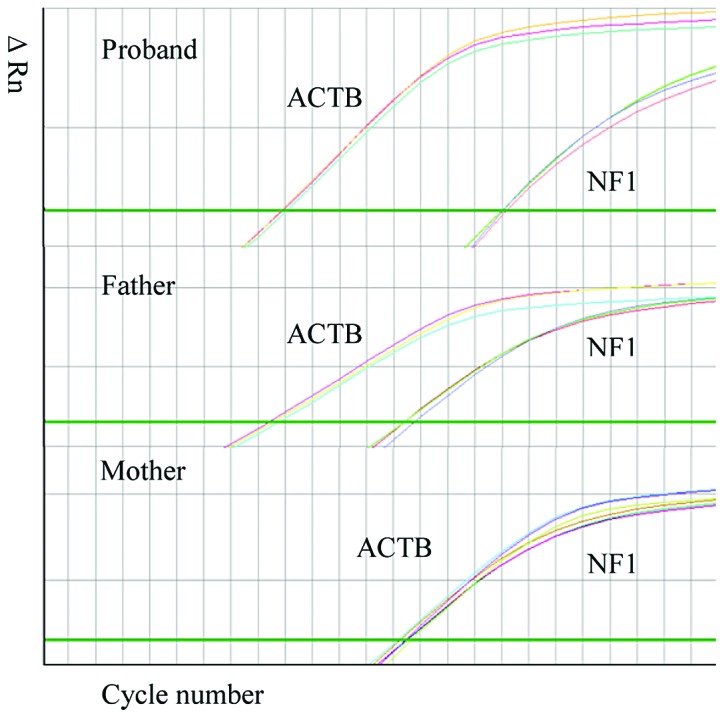
Real-time PCR profiles of exon 20 skipping in the family with the germline mutation c.3362A>G found in the son and father. As the template amount decreased, the cycle number increased in the amplification plot. The data indicated that exon 20 template was less in the sample of the son.

Of note, one exonic mutation, c.3362A>G, produced mosaicism of E1121G and exon 20 skipping at the mRNA level (Fig. 1). This germline mutation in the proband was inherited from his father, and the post-transcriptional mosaicism was presented in the mRNA of both patients. However, the missense mutation of the son showed a lower signal intensity using Sanger sequencing (Fig. 2). The mosaicism of the post-transcriptional profile was further examined using real-time PCR with cDNA obtained from this family (Fig. 3). The sample from the mother was used as the wild-type sample in this assay. The skipping rate of the mutant exon in the samplefrom the son was higher compared to the rate in the sample from the father.

### In silico analysis

To better understand the underlying mechanisms of these 16 unusual splicing errors, 7 computational tools were employed to examine how these mutations affected the splicing-relative sequence motifs. The comparison was made between the results of the prediction on the normal and mutated sequences (Table IV). SSPNN predicted a deletion of the authentic acceptor site (3′ss) caused by 2410-18C>G, and a decrease in the strength of the authentic splice sites caused by 1260+3A>G and 5944-5A>G in the subgroup I. By contrast, the cryptic splice sites generated by these 3 mutations and the subgroup II mutations were given high scores by HSF or SSPNN. Substitutions in the subgroup III all abolished the authentic donor sites (5′ss) in the SSPNN evaluation, compared to the prediction of the strength reduction by HSF. No cryptic splice sites were predicted in these substitution sequences and the subgroup IV sequences.

Further investigation on other splicing-relative sequence motifs revealed that some cis-acting factors were altered in these mutated sequences. In the subgroup I sequences, the branch-point motif was deleted and a putative ESE was generated by 2410-18C>G; a putative ESS or an abolished ESS was predicted to accompany the strength-decreased donor site or acceptor site in the mutated sequences of 1260+3A>G and 5944-5A>G, respectively. In the subgroup II sequences, the ESE was deleted or the strength of the ESE was decreased simultaneously with the strong new splice site generated by 1466A>G and 1885G>A, respectively. The original ESE was deleted or no ESE was embedded in the subgroup III sequences. The architectural alterations of the splicing-regulatory elements were more complex in the subgroup IV sequences. In general, the decreased strength of the ESE or decreased ratio of the ESE/ESS was presented. An abolished ESS was also predicted in mutated exon 7 and 37, but no abolished ESE was predicted.

## Discussion

NF1 is a multisystem genetic disorder with extreme diversity of clinical expression (3,10,11,20,21,22). A clear correlation of genotype and phenotype has been previously demonstrated in only two types of mutations (23). Patients with an *NF1* microdeletion have more severe clinical characteristics (4,23). We found 5 cases with an *NF1* gene deletion in patients with various severe conditions, such as developmental delay, seizures, and early onset skin/subcutaneous tissue disorders. One 4 year-old patient harboring a novel splice site mutation (60+1delG) also exhibited the developmental delay. This alternation, which induced skipping in exon 1 may cause the same effect as an *NF1* deletion at the protein level. Another mutation, c.2970-2972delAAT, in exon 17, which was not shown in our study, has been reported to be associated with the absence of cutaneous neurofibromas (11,24). However, due to the lack of detailed clinical information, our investigation of the correlation between genotype and phenotype was limited. The present study focused largely on the mutation spectrum, particularly the splicing errors.

The mutation rate of the *NF1* gene is one of the highest reported in the human genome. We presented a mutation spectrum of the *NF1* gene with 127 different variants in this study. In the summarized data, exon 19b, 25 and 29 harbored more missense mutations, exon 4b, 10a and 23.2 appeared to have more nonsense mutations, and the highest rate of frameshift mutations was found in exon 28. In addition, exon 7, 20 and 37 were prone to splicing errors. A total of 54 of the 129 different mutations were novel mutations that were categorized as either frameshift mutations, splicing defects, nonsense mutations or missense mutations. With the exception of missense mutations, the other 3 types of mutations are considered highly likely to be deleterious. Patients with NF1 with a missense mutation have a lower incidence of multiple neurofibromas and plexiform neurofibromas compared to patients with a different type mutation; In addition, it is also true that no evidently milder NF1 phenotype was concluded to be distinctly associated with a missense mutation (23). The *in silico* analysis of the functional consequence of these novel missense mutations was performed using the Polyphen2 and SIFT program. A total of 12 of these 14 novel missense mutations were predicted to be potentially damaging. Although P1830L and A1952V were determined to be benign, these mutations and other novel missense mutations altered amino acids that were conserved across the different species according to the results obtained from an orthologous alignment using BLASTP. It is widely believed that mutations in a highly conserved region may result in a functional change. Moreover, we found 2 second missense variants, M645V and I1658V, with nonsense mutations. These 2 variants can be treated as neutral polymorphism after the parental study.

In the present study, 25.2% of the different mutations induced aberrant splicing and 50% of these splicing errors were caused by exonic mutations and intronic mutations at non-consensus splice sites. These results are consistent with those of previous findings, showing that the *NF1* gene is susceptible to having splicing errors in post-transcription (14,15). Accurate splice site recognition is critical in pre-mRNA splicing (25). This process is a coordinated program involving the strong splice sites, correct splicing regulatory elements (SRE) embedded in the genome and the associated proteins. Bioinformatics assessments focused on revealing how the authentic splice sites and the hidden sequence motifs of these SREs were interfered by these exonic and intronic variants at non-consensus splice sites in this study. We found that the change in these splice sites acted by tethering the alteration of the ESE, ESS and other cis-acting elements, which resulted in aberrant splicing. The error-prone splicing occurred in the subgroups I and II in which the high-score cryptic 5′/3′ss or the next AG/GT with a higher score, as the case of 1260+3A>G, replaced the strength-decreased or lower-scored authentic ones in the new microenvironment of splicing regulatory elements. For example, the 2410-18C>G mutation generated a higher score cryptic splice site while forming a putative ESE and abolishing the original branch point, and then 17 base pairs were inserted into the mRNA as the consequences of a strong cryptic splice site in coordination with the gain and/or loss of SREs. Importantly, the 2410-16A>G, 2410-15A>G, 2410-12T>G mutations have been previously documented in the Biobase HGMD database. Given 2410-18C>G, it is an obvious sign to alert that this cluster near the intron 15/exon 16 boundary is a splicing-aberration harbor.

In some cases, the disruption of ESE elements was the principal cause of the splicing error due to the consequences of the reduced splicing enhancement activity (16,26,27). As is known, not all of the substitutions at the last nucleotide position of an exon will cause exon skipping, although the mutants are also predicted to delete the authentic splice site or decrease its strength. The loss of ESE motifs may explain why the exon skipping was caused by the exonic mutations in the subgroup III. An exception in this case was exon 29 that had no ESE motif. Exon 29 skipping resulted from the weakened donor site caused by the mutation 5546G>A and the lack of the ESE to support the splice-site recognition.

There was no cryptic splice site generated and no strength reduction of the authentic splice sites in the subgroup IV sequences. However, the acceptor site strength was characterized to be an important and sensitive parameter in splice-site recognition (28). The low-score exons, such as exon 7 and 37 were observed to have more splicing defects in this study. In addition, our *in silico* analysis also showed more complex changes in the ESE and ESS. Not only was the ESE demonstrating a decrease in strength, but it also showed that weaker ESS, decreased ratio of ESE/ESS and abolished ESS were predicted to be the architectural weakness in the exon definition, resulting in exon exclusion in the subgroup IV sequences. The ratio of ESE/ESS was important for exon recognition and intron identification in the complexity of splicing. Several studies have observed that a higher density of ESEs was in the exons compared to the introns and vice versa for ESSs (12,29,30). The ratio decreasing of ESE/ESS will break the delicate balance of SREs, and will be prone to exon exclusions. Although enhancers and silencers have apparently opposite effects as suggested by their terms, the Composite Exonic Regulatory Elements of Splicing (CERES) has already been proposed when accumulating evidence has suggested ESE and ESS shared additional properties (31,32). The findings of the weaker or abolished ESS caused by the mutations in our analysis reflected the overlapping function of these 2 elements. The SREs became more critical in determining the splice-site recognition in these cases.

When the trans-acting factors navigated the new landscape in which the mutation plays a make-or-break role, the consequences of the competition and coordination in the splicing process is more evident in the case with the 3362A>G mutation, in which the germline mutation decreased the ratio of the ESE/ESS. The splicing in the mutated microenvironment resulted in the missense mutation and exon skipping coexisting in the mRNA of an 11-year-old boy and his father with different rates. Furthermore, real-time PCR clearly confirmed this mosaicism. Both patients have multiple café-au-lait spots but no other profoundly different and NF1-related clinical features. The higher skipping rate found in the sample of the son may be caused by individual’s genetic variability.

Taken together, this study presents 54 novel *NF1* mutations and reveals the high frequency of the unusual splicing defects in the pathogenicity of NF1. Integrated analyses using the bioinformatics tools provided insight in order to better understand the underlying mechanisms of these splicing errors. In particular, as the *NF1* gene was susceptible to having aberrant splicing caused by exonic variants, including the silent mutation, such as 1185G>A, this result underscored the large consequences of *NF1* gene testing at both the genomic and mRNA levels. In addition, the mutation data may contribute information to the ongoing antisense therapeutics for NF1 caused by intronic mutations (33), thus shedding light on a targeted treatment to restore *NF1* gene function.

## Figures and Tables

**Table I:** The 127-mutation spectrum apart from the splicing abnormalities.

cDNA	Protein	Exon
Missense mutations		
1A>G	Met1Val	1
**410C>G**	**Ser137Cys**	4a
1241T>G	Leu414Arg	9
1646T>C	Leu549Pro	11
2350 T>C	Trp784Arg	15
**2975T>A**	**Met992Lys**	17
**3104T>C**	**Met1035Thr**	18
3142T>G	Trp1048Gly	19a
**3211G>C**	**Ala1071Pro**	19b
3295A>G	Lys1099Glu	19b
**3296A>C**	**Lys1099Thr**	19b
**3362A>G**	**Glu1121Gly**	20
3494T>C	Ile1165Thr	20
**3752A>C**	**His1251Pro**	22
3827G>A	Arg1276Gln	22
**4172G>C**	**Arg1391Thr**	24
4288A>T	Asn1430Tyr	25
4306A>G	Lys1436Glu	25
4306A>C	Lys1436Gln	25
**4318A>G**	**Met1440Val**	25
4493G>A	Gly1498Glu	26
**5131G>C**	**Ala1711Pro**	28
5425C>T	Arg1809Cys	29
**5489C>T**	**Pro1830Leu**	29
**5498T>C**	**Leu1833Pro**	29
**5855C>T**	**Ala1952Val**	31
**7106T>C**	**Leu2369Ser**	39
Nonsense mutations		
**569T>G**	Leu190^*^	4b
574C>T	Arg192^*^	4b
**586G>T**	Glu196^*^	4b
668G>A	Trp223^*^	5
1238C>G	Ser413^*^	9
1246C>T	Arg416^*^	9
1275G>A	Trp425^*^	10a
1318C>T	Arg440^*^	10a
1381C>T	Arg461^*^	10a
1754T>A	Leu585^*^	12a
2041C>T	Arg681^*^	13
2352G>A	Trp784^*^	15
2446C>T	Arg816^*^	16
3049C>T	Gln1017^*^	18
3826C>T	Arg1276^*^	22
4006C>T	Gln1336^*^	23.2
**4066G>T**	Glu1356^*^	23.2
4084C>T	Arg1362^*^	23.2
4107C>G	Tyr1369^*^	23.2
**4267A>T**	Lys1423^*^	24
4537C>T	Arg1513^*^	27a
5264C>G	Ser1755^*^	29
5401C>T	Gln1801^*^	29
**5708T>G**	Leu1903^*^	30
5839C>T	Arg1947^*^	31
5941C>T	Gln1981^*^	31
**6243C>G**	Tyr2081^*^	33
6709C>T	Arg2237 ^*^	36
7285C>T	Arg2429^*^	41
7486C>T	Arg2496^*^	42
7843C>T	Gln2615^*^	45
7993C>T	Gln2665^*^	46
**8072G>A**	Trp2691^*^	47
Frameshift mutations		
**118delA**	Lys40Argfs^*^4	2
**154delT**	Ser52euLfs^*^4	2
499_502delTGTT	Cys167Glnfs^*^12	4b
**802_803delCCinsG**	Pro268Aspfs^*^13	6
**1010_1021del12**	**Glu337_Ser340del**	7
1541_1542delAG	Gln514Argfs^*^43	10c
**1664_1667delTAGA**	556Aspdelfs^*^13	11
1756_1759delACTA	Phe586delfs^*^19	12a
1882delT	Tyr628Thrfs^*^3	12b
1908delT	Ser636Valfs^*^52	12b
**2032_2033delCCinsA**	Pro678Lysfs^*^10	13
**2342dupA**	His781Glnfs^*^13	15
**2984_2988delTGGTC**	Leu995Glnfs^*^24	17
**3054delT**	Leu1018^*^	18
**3108_3109ins23**	Lys1036Thrfs^*^8	18
**3852_3854delAAT**	**Lle1284fs**	22
4312_4314delGAA	Glu1438del	25
4418_4419delAT	His1473Glnfs^*^7	26
**4498_4505delTATCTTTC**	Tyr1500_Ile1501delfs^*^6	26
**4688_4689insAA**	Phe1563Leufs^*^5	27b
**4810dupT**	Tyr1604Leufs^*^16	28
**4906dupA**	Asp1636Argfs^*^5	28
**4930_4937indels**	Val1644Serfs^*^3	28
5010delG	Lys1670Asnfs^*^7	28
**5040_5043delAGGTinsTA**	Lys1680Asnfs^*^16	28
**5909dupC**	Ile1971Tyrfs^*^17	31
6791dupA	Tyr2264^*^	37
7096_7101delAACTTT	Asn2366_Phe2367del	39
7125delA	Tyr2375Thrfs^*^20	39
**7160delG**	Arg2387Lysfs^*^10	40
7267dupA	Thr2423Asnfs^*^4	41
**7388_7384+12del19**	Unknown	41
**7510delG**	Asp2504Thrfs^*^23	42
**7581_7582delAT**	Ser2528Glnfs^*^21	43
**7726delT**	Ser2576Glnfs^*^27	44

Novel mutations were denoted in bold text.

**Table II:** The mutation spectrum of the splicing abnormalities.

Splicing mutations at the consensus splice sites			

Mutation	IVS	cDNA effect	Effect on splice site
**60+1delG**	**IVS1+1delG**	Unknown	Inactive 5′ss
204+1G>A	IVS2+1G>A	100_204del105	Cryptic 5′ss
205-2A>C	IVS2-2A>C	ΔE3	Inactive 3′ss
889-2A>G	IVS6-2A>G	ΔE7	Inactive 3′ss
889-1G>A	IVS6-1G>A	ΔE7	Inactive 3′ss
1642-1G>A	IVS10c-1G>A	ΔE11	Inactive 3′ss
2409+1G>A	IVS15+1G>A	ΔE15	Inactive 5′ss
2850+1G>A	IVS16+1G>A	ΔE16	Inactive 5′ss
3114-2A>G	IVS18-2A>G	ΔE19a	Inactive 3′ss
3708+2T>A	IVS21+2T>A	ΔE21	Inactive 5′ss
3709-2A>G	IVS21-2A>G	3709_3718delGATGAACTAG	Cryptic 3′ss
4367+1G>A	IVS25+1G>A	ΔE25	Inactive 5′ss
6579+1G>A	IVS34+1G>A	ΔE34	Inactive 5′ss
6858+1G>A	IVS37+1G>A	ΔE37	Inactive 5′ss
7676-2A>G	IVS43-2A>G	ΔE44	Inactive 3′ss
8098-1G>A	IVS47-1G>A	ΔE48	Inactive 3′ss

Intronic mutations at non-consensus splice sites			

Mutation	Related IVS	cDNA effect	Cryptic splice site

**1260+3A>T**	**IVS9+3A>T**	**1261_1262insGTTAGTCCAAAAG**	Cryptic 5′ss
**2410-18C>G**	**IVS15-18C>G**	**2410_2411ins17**	Cryptic 3′ss
5944-5A>G	IVS31-5A>G	5943_5944insCTAG	Cryptic 3′ss

Exonic mutations			

Mutation	Related exon	cDNA effect	Cryptic splice site

910C>T	In E7	ΔE7	No
**972T>A**	In E7	ΔE7	No
**1185G>A**	Last NT of E8	ΔE8	No
1466A>G	In E10b	1466_1572del62	Cryptic 5′ss
**1720A>G**	Last second NT of E11	ΔE11	No
1885G>A	In E12b	1846_1886del	Cryptic 3 ′ss
**3362A>G**	In E20	ΔE20	No
**3467A>G**	In E20	ΔE20	No
**3496G>A**	Last NT of E20	ΔE20	No
5546G>A	Last NT of E29	ΔE29	No
6792C>A	In E37	ΔE37	No
6792C>G	In E37	ΔE37	No
**7694delC**	In E44	ΔE44	No

Novel mutations were denoted in bold text. ss, splice sites.

**Table III:** The number of each type of abnormality in 127 mutations and the frequency of the novel mutations.

	Missense	Nonsense	Deletion	Ins/Dup	Indels	Splicing defect
Total no.	27	33	23	8	4	32
Novel mutations	14	7	13	6	4	10

**Table IV:** The scores and results assessed using the computational tools on the normal and mutant sequences.

	Predicted scores by HSF			Predicted scores by SSPNN		Prediction
			
Germline mutations	Authentic ss on normal sequences	Authentic ss on mutant sequences	New ss generated	Authentic ss on normal sequences	Authentic ss on mutant sequences	ESE, ESS and other motifs on mutant sequences
Subgroup I: Intronic mutation						
1260+3A>T	Donor site (88.18)	Donor site (83.15)	Donor site (79.6)	Donor site (0.99)	Donor site (0.80)	Putative ESS
2410-18C>G	Acceptor site (84.37)	Acceptor site (84.37)	Acceptor site (86.14)	Acceptor site (0.46)	Acceptor site (none)	Putative ESE, abolished BP
5944-5A>G	Acceptor site (80.89)	Acceptor site (80.83)	Acceptor site (92.26)	Acceptor site (0.78)	Acceptor site (0.74)	Abolished ESS
Subgroup II: Exonic mutation with cryptic ss						
1466A>G	Donor site (82.29)	Donor site (82.29)	Donor site (82.62)	Donor site (none)	Donor site (0.97)	Abolished ESE
1885G>A	Acceptor site (82.35)	Acceptor site (82.35)	Acceptor site (94.06)	Acceptor site (none)	Acceptor site (0.98)	ESE and ESS strength decreased
Subgroup III: Substitution at last NT or last 2nd NT of an exon						
1185G>A	Donor site (95.55)	Donor site (84.97)	No	Donor site (0.99)	Donor site (0.51)	Abolished ESE
1720A>G	Donor site (77.05)	Donor site (72.19)	No	Donor site (0.79)	Donor site (none)	Abolished ESE
3496G>A	Donor site (94.52)	Donor site (71.5)	No	Donor site (0.79)	Donor site (none)	Abolished ESE
5546G>A	Donor site (85.96)	Donor site (75.38)	No	Donor site (0.97)	Donor site (none)	No original ESE
Subgroup IV: Exonic mutation without cryptic ss						
910C>T	-	-	No	-	-	Abolished ESS
972T>A	-	-	No	-	-	ESE strength decreased
3362A>G	-	-	No	-	-	ESE/ESS ratio decreased
3467A>G	-	-	No	-	-	ESS strength decreased
6792C>A/G	-	-	No	-	-	Abolished ESS
7694delC	-	-	No	-	-	ESE/ESS ratio decreased

The cut off value set by SSPNN was 0.40. The unchanged scores valued for the exonic mutations without cryptic splice sites were denoted by the sign ‘-’. HSF, Human Splicing Finder; SSPNN, Splice Site Prediction by Neural Network; ESE, exonic splicing enhancer; ESS, exonic splicing silencer; ss, splice sites.

## References

[1] Williams VC, Lucas J, Babcock MA, Gutmann DH, Korf B, Maria BL (2009). Neurofibromatosis type 1 revisited. Pediatrics.

[2] (1988). Neurofibromatosis. Conference statement. National Institutes of Health Consensus Development Conference. Arch Neurol.

[3] Griffiths S, Thompson P, Frayling I, Upadhyaya M (2007). Molecular diagnosis of neurofibromatosis type 1: 2 years experience. Fam Cancer.

[4] Pasmant E, Sabbagh A, Spurlock G, Laurendeau I, Grillo E, Hamel MJ, Martin L, Barbarot S, Leheup B, Rodriguez D, Lacombe D, Dollfus H, Pasquier L, Isidor B, Ferkal S, Soulier J, Sanson M, Dieux-Coeslier A, Bièche I, Parfait B, Vidaud M, Wolkenstein P, Upadhyaya M, Vidaud D, members of the NF France Network (2010). NF1 microdeletions in neurofibromatosis type 1: from genotype to phenotype. Hum Mutat.

[5] Aoki Y, Niihori T, Narumi Y, Kure S, Matsubara Y (2008). The RAS/MAPK Syndromes: Novel roles of the RAS pathway in human genetic disorders. Hum Mutat.

[6] Cichowski K, Jacks T (2001). NF1 tumor suppressor gene function: narrowing the GAP. Cell.

[7] Yunoue S, Tokuo H, Fukunaga K, Feng L, Ozawa T, Nishi T, Kikuchi A, Hattori S, Kuratsu J, Saya H, Araki N (2003). Neurofibromatosis type I tumor suppressor neurofibromin regulates neuronal differentiation via its GTPase-activating protein function toward Ras. J Biol Chem.

[8] Schubbert S, Shannon K, Bollag G (2007). Hyperactive Ras in developmental disorders and cancer. Nat Rev Cancer.

[9] Trovó-Marqui AB, Tajara EH (2006). Neurofibromin: a general outlook. Clin Genet.

[10] Nemethova M, Bolcekova A, Ilencikova D, Durovcikova D, Hlinkova K, Hlavata A, Kovacs L, Kadasi L, Zatkova A (2013). Thirty-nine novel neurofibromatosis 1 (NF1) gene mutations identified in Slovak patients. Ann Hum Genet.

[11] Ko JM, Sohn YB, Jeong SY, Kim HJ, Messiaen LM (2013). Mutation spectrum of NF1 and clinical characteristics in 78 Korean patients with neurofibromatosis type 1. Pediatr Neurol.

[12] Zhang C, Li WH, Krainer AR, Zhang MQ (2008). RNA landscape of evolution for optimal exon and intron discrimination. Proc Natl Acad Sci USA.

[13] Kolovos P, Knoch TA, Grosveld FG, Cook PR, Papantonis A (2012). Enhancers and silencers: an integrated and simple model for their function. Epigenetics Chromatin.

[14] Messiaen LM, Callens T, Mortier G, Beysen D, Vandenbroucke I, Van Roy N, Speleman F, Paepe AD (2000). Exhaustive mutation analysis of the NF1gene allows identification of 95% of mutations and reveals a high frequency of unusual splicing defects. Hum Mutat.

[15] Ars E, Serra E, Garcia J, Kruyer H, Gaona A, Lázaro C, Estivill X (2000). Mutations affecting mRNA splicing are the most common molecular defects in patients with neurofibromatosis type 1. Hum Mol Genet.

[16] Zatkova A, Messiaen L, Vandenbroucke I, Wieser R, Fonatsch C, Krainer AR, Wimmer K (2004). Disruption of exonic splicing enhancer elements is the principal cause of exon skipping associated with seven nonsense or missense alleles of NF1. Hum Mutat.

[17] Wimmer K, Roca X, Beiglböck H, Callens T, Etzler J, Rao A, Krainer A, Fonatsch C, Messiaen L (2007). Extensive in silico analysis of NF1 splicing defects uncovers determinants for splicing outcome upon 5′ splice-site disruption. Hum Mutat.

[18] Pros E, Gómez C, Martín T, Fábregas P, Serra E, Lázaro C (2008). Nature and mRNA effect of 282 different NF1 point mutations: focus on splicing alterations. Hum Mutat.

[19] Sironi M, Menozzi G, Riva L, Cagliani R, Comi GP, Bresolin N, Giorda R, Pozzoli U (2004). Silencer elements as possible inhibitors of pseudoexon splicing. Nucleic Acids Res.

[20] Ars E, Kruyer H, Morell M, Pros E, Serra E, Ravella A, Estivill X, Lázaro C (2003). Recurrent mutations in the NF1 gene are common among neurofibromatosis type 1 patients. J Med Genet.

[21] Castle B, Baser ME, Huson SM, Cooper DN, Upadhyaya M (2003). Evaluation of genotype-phenotype correlations in neurofibromatosis type 1. J Med Genet.

[22] Kluwe L, Friedrich R, Korf B, Fahsold R, Mautner V (2002). NF1 mutations in neurofibromatosis 1 patients with plexiform neurofibromas. Hum Mutat.

[23] van Minkelen R, van Bever Y, Kromosoeto J, Withagen-Hermans C, Nieuwlaat A, Halley D, van den Ouweland A (2013). A clinical and genetic overview of 18 years neurofibromatosis type 1 molecular diagnosis in the Netherlands. Clin Genet.

[24] Upadhyaya M, Huson SM, Davies M, Thomas N, Chuzhanova N, Giovannini S, Evans DG, Howard E, Kerr B, Griffiths S, Consoli C, Side L, Adams D, Pierpont M, Hachen R, Barnicoat A, Li H, Wallace P, Van Biervliet JP, Stevenson D, Viskochil D, Baralle D, Haan E, Riccardi V, Turnpenny P, Lazaro C, Messiaen L (2007). An absence of cutaneous neurofibromas associated with a 3-bp inframe deletion in exon 17 of the NF1 gene (c.2970–2972 delAAT): evidence of a clinically significant NF1 genotype-phenotype correlation. Am J Hum Genet.

[25] Nelson KK, Green MR (1990). Mechanism for cryptic splice site activation during pre-mRNA splicing. Proc Natl Acad Sci USA.

[26] Blencowe BJ (2000). Exonic splicing enhancers: mechanism of action, diversity and role in human genetic diseases. Trends Biochem Sci.

[27] Colapietro P, Gervasini C, Natacci F, Rossi L, Riva P, Larizza L (2003). NF1 exon 7 skipping and sequence alterations in exonic splice enhancers(ESEs) in a neurofibromatiosis 1 patient. Hum Genet.

[28] Vandenbroucke I, Callens T, De Paepe A, Messiaen L (2002). Complex splicing pattern generates great diversity in human NF1 transcripts. BMC Genomics.

[29] Wang Z, Rolish ME, Yeo G, Tung V, Mawson M, Burge CB (2004). Systematic identification and analysis of exonic spicing silencers. Cell.

[30] Zhang XH, Chasin LA (2004). Computational definition of sequence motifs governing constitutive exon splicing. Genes Dev.

[31] Baralle D, Baralle M (2005). Splicing in action: assessing disease causing sequence changes. J Med Genet.

[32] Haque A, Buratti E, Baralle FE (2010). Functional properties and evolutionary splicing constraints on a composite exonic regulatory element of splicing in CFTR exon 12. Nucleic Acids Res.

[33] Gottfried O, Viskochil D, Couldwell W (2010). Neurofibromatosis type 1 and tumorigenesis: molecular mechanisms andtherapeutic implications. Neurosurg Focus.

